# Supplementary material for: Imputation of ancient human genomes
Source: Nat Commun. 2023 Jun 20;14:3660. doi: 10.1038/s41467-023-39202-0 (PMC10282092; doi:10.1038/s41467-023-39202-0)
Supplement: Supplementary file 1 — Supplementary Information [file 41467_2023_39202_MOESM1_ESM.pdf]

Supplementary information

Bárbara Sousa da Mota[1,2], Simone Rubinacci[1,2], Diana Ivette Cruz Dávalos[1,2], Carlos Eduardo G. Amorim[3], Martin Sikora[4], Niels N. Johannsen[5], Marzena Szmyt[6], Piotr Włodarczak[7], Anita Szczepanek[7,8], Marcin M. Przybyła[9], Hannes Schroeder[10], Morten E. Allentoft[11,4], Eske Willerslev[4,12,13,14], Anna-Sapfo Malaspinas[1,2]*@, Olivier Delaneau[1,2]*

[1]Department of Computational Biology, University of Lausanne, Switzerland

[2]Swiss Institute of Bioinformatics, University of Lausanne, Switzerland

[3]Department of Biology, California State University, Northridge, California, United States of America

[4]Lundbeck Foundation GeoGenetics Centre, Globe Institute, University of Copenhagen, Copenhagen, Denmark

[5]Department of Archaeology and Heritage Studies, Aarhus University, Denmark

[6]Institute for Eastern Research, Adam Mickiewicz University in Poznań, Poznań, Poland

[7]Institute of Archaeology and Ethnology, Polish Academy of Sciences, Kraków, Poland

[8]Department of Anatomy, Jagiellonian University, Medical College, Kraków, Poland

[9]Institute of Archaeology, Jagiellonian University, Kraków, Poland

[10]The Globe Institute, Faculty of Health and Medical Sciences, University of Copenhagen, Copenhagen, Denmark

[11]Trace and Environmental DNA (TrEnD) Laboratory, School of Molecular and Life Science, Curtin University, Australia

[12]GeoGenetics Group, Department of Zoology, University of Cambridge, Cambridge, UK

[13]Wellcome Sanger Institute, Wellcome Genome Campus, Cambridge, UK

[14]MARUM, University of Bremen, Bremen, Germany

* These authors jointly supervised this work

Supplementary Note 1: Effect of using different genotype callers to generate genotype likelihoods, i.e., input for imputation

In order to determine whether the choice of genotype caller to calculate genotype likelihoods prior to imputation influences the quality of the imputed calls, we compared the accuracy of imputation using genotype likelihoods obtained with two different callers i) bcftools[1], a tool designed to handle modern DNA, and ii) ATLAS[2], a genotype caller that models and/or estimates deamination patterns and takes these into account when calling genotypes for ancient genomes. In the case of ATLAS, we empirically estimated the damage pattern before proceeding to genotype calling. In addition, to compute the imputation accuracy, we used two different validation sets, where calls were obtained using i) bcftools and ii) ATLAS. Supplementary Fig. 1 shows imputation accuracy obtained with the four previously described configurations for a subset of 16 genomes downsampled to 1.0x prior to imputation. For a few genomes, such as Lovelock2[3] and SIII[4], there were no noticeable differences between the different configurations. In most cases, the most striking differences were between validation sets, regardless of the genotype likelihoods set used for imputation. Indeed, the accuracy curves tend to cluster by validation rather than genotype likelihood set. However, in the case of Sumidouro5, there is a larger difference between the two different genotype likelihood configurations for the same validation set, particularly at sites with minor allele frequency (MAF) below 5%. For this genome, with 40% frequency of C-to-T substitutions at the reads' ends, the highest accuracy was obtained with both imputation using genotype likelihoods and validation calls obtained with ATLAS. In conclusion, imputation calls were not significantly affected by the choice of tool to call genotype likelihoods for most of the cases here analyzed and, therefore, we chose to calculate genotype likelihoods using bcftools with no further filtering before imputation. However, the two genotype callers used to obtain the validation calls from the high-coverage genomes had clear differences and we further investigated the differences between them in the next section.

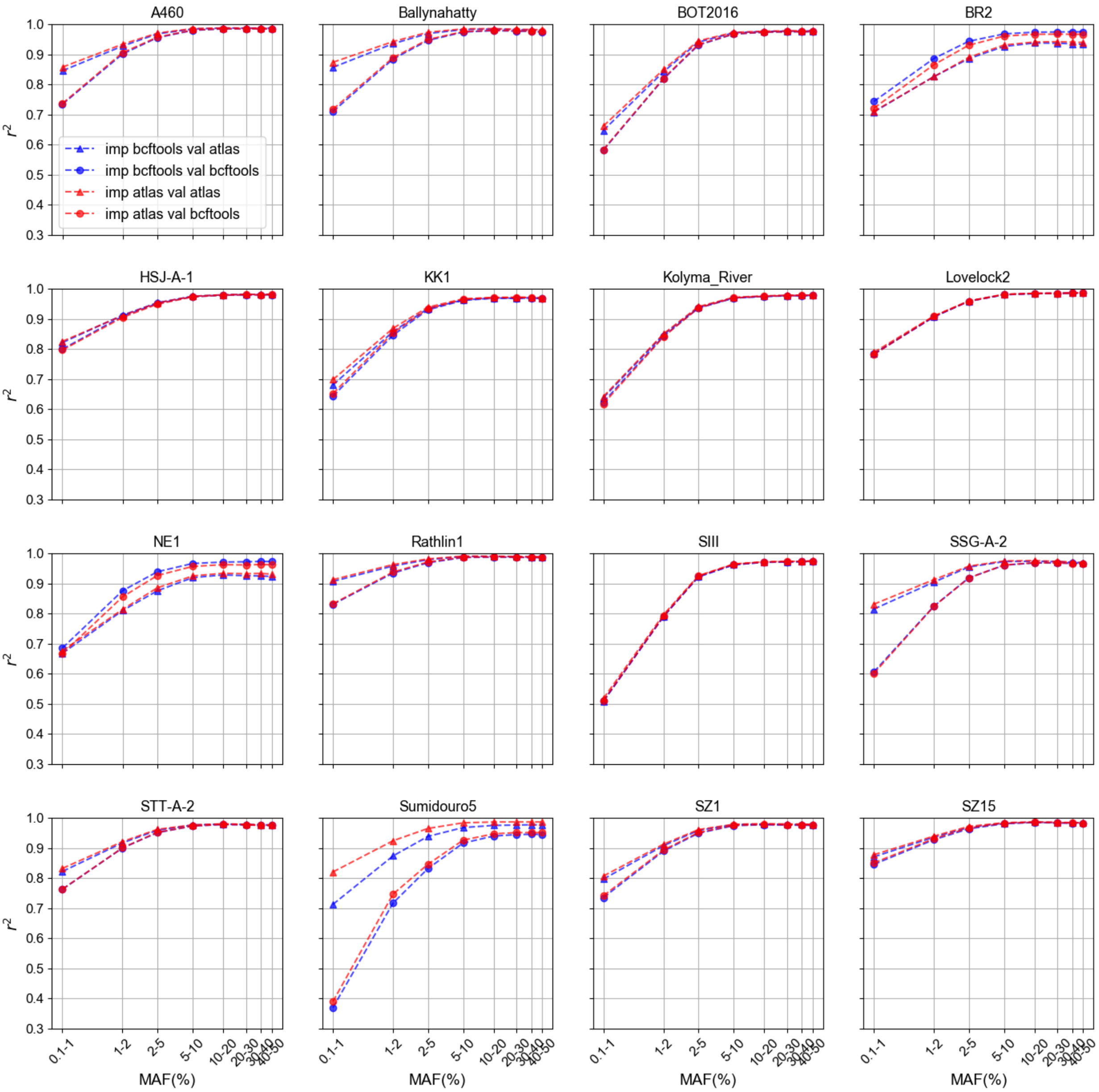

Supplementary Fig. 1. Effect of genotype caller on imputation accuracy. Imputation accuracy for a subset of 16 imputed 1.0x genomes, where imputation was performed from genotype likelihoods calculated with i) bcftools (purple) and ii) ATLAS (green). Two different validation sets were used in these analyses that differ in the tool used to call genotypes: i) bcftools (circles) and ii) ATLAS (triangles).

Supplementary Note 2: Validation dataset

In order to be able to assess imputation accuracy when imputing low-coverage ancient genomes, we resorted to downsampling and imputing ancient genomes with average depth of coverage above 10x, using the high-coverage genomes as ground truth. However, these high-coverage genomes are not free from the inherent ancient DNA challenges, in particular, base deamination. This constitutes a problem in determining what the true genotypes are for a particular genome, thus affecting how well we can validate imputation results in the context of this work. To determine how we can best circumvent this problem, we tried different approaches to generate the validation calls that were expected to decrease the impact of ancient DNA damage on the resulting genotype calls in the case of four 1x ancient genomes, namely, RISE1168[5,6], SIII[4] (UDG-treated), Sumidouro5[3] (a highly damaged genome) and WC1[7] (with intermediately high damage levels). Firstly, we called genotypes with ATLAS, a genotype caller that models the deamination patterns at the ends of reads, except for RISE1168, that includes paired-end libraries in addition to single-end libraries. Then, prior to genotype calling with bcftools, we trimmed five base pairs from the reads in the bam files using BamUtil[8]. We also followed the filtering approach carried out in previous studies[3]: i) we called genotypes with bcftools using reads with mapping quality of at least 30 and bases with quality 20 ("-q 30 -Q 20"), as well as the recommended option -C 50; ii) we retained only the sites present in the 1000 Genomes accessible genome strict mask; iii) we excluded sites located in regions known to contain repetitions; iv) we removed sites that have depth below the maximum of one third of the mean depth of coverage and eight, as this is typically the minimum depth of coverage for which we can confidently call genotypes, and sites that have depth above twice the mean depth; v) we retained sites for which the field QUAL is equal or greater than 30. Finally, we used these different genotype call sets as validation when evaluating imputation accuracy for the four aforementioned genomes. We learnt that the application of the aforementioned five filters gives a consistently higher agreement between imputation and validation calls, even though it does not yield the highest accuracy for the four genomes (Supplementary Fig. 2). In the case of Sumidouro5, ATLAS outperformed the other approaches, with the five filters approach in second place, but

ATLAS performance was not consistent across samples. The trimming approach yielded intermediate accuracy curves, in general, but, compared to the five-filter option in the case of Sumidouro5, it yielded much lower values at rare variants (MAF<2%). Given these results, we chose to generate the validation calls used in all the analyses in the main paper by applying the five-filter approach. However, we are aware that more accurate calls could be obtained by applying stricter filters for particular genomes that contain more degradation, including Sumidouro5, as was done in Moreno-Mayar et al.[3] We decided, instead, to apply the same approach to all 42 high-coverage genomes to expedite the process and have consistent datasets.

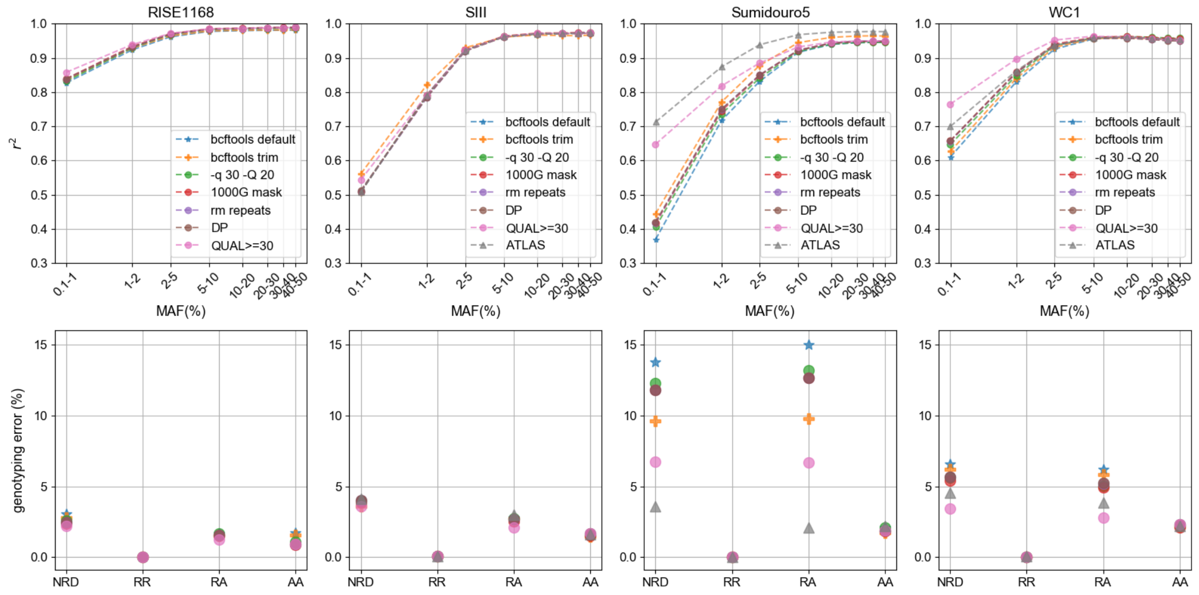

Supplementary Fig. 2. Effect of applying quality filters to validation call set on imputed genotypes. Imputation accuracy (first row) and genotype discordance (second row) for four different genomes, organized by column (RISE1168[5,6], SIII[4], Sumidouro5[3] and WC1[7]). The imputed genomes were downsampled to 1x coverage. The filters were applied in the following order: i) genotype calling with bcftools using reads with mapping quality of at least 30 and base quality 20 ("-q 30 -Q 20"), as well as the recommended option -C 50; ii) only sites present in the 1000 Genomes strict mask are retained; iii) exclusion of sites that are located in regions known to contain repetitions; iv) filtering out of sites that have depth below the maximum of one third of the mean depth of coverage and eight and sites that have depth above twice the mean depth; v) retaining

sites for which the field QUAL is equal or greater than 30. For each validation and filter, the same imputed data were used in this analysis.

Supplementary Note 3: Individual samples

Supplementary Table 1. Information on the ancient genomes used in the benchmark of imputation of low-coverage genomes. Id, modern country where remains were found, age of remains in years before present (yBP), depth of coverage as reported in the original publications and at the 1000 Genomes bi-allelic sites, frequency of C-to-T mismatches at the 5' end (if entry contains "*", the value or label is the one reported in the publication; if entry contains "-", it means we could not properly estimate the damage frequency and it was not reported in the publication in a clear way; otherwise, the values were obtained with bamdamage[9]), population of the 1000 Genomes panel whose minor allele frequency (MAF) was used in imputation accuracy analyses for each of the individuals, when applicable (AFR: Africa, AME: America, EAS: East Asia, EUR: Europe, SAS: South East Asia, All: overall allele frequency in 1000 Genomes), and respective publication. For RISE1160, a low-coverage genome, we do not indicate a MAF label, as we did not estimate imputation accuracy as a function of MAF for this genome. In the case of the ancient trio age (RISE1159, RISE1160 and RISE1168), we report a time span obtained for the mass grave as a whole. This range is the result of a model that took into consideration the number of contemporaneous individuals in the grave, radiocarbon dating of the different remains, as well as the ontogenetic constraints on how much the ages (e.g., parent/offspring relations) can vary[5].

ID	Country	Age range (yBP)	Reported coverage	Coverage in 1000G	C->T	MAF label	Study
atp016	Spain	4867-5212	13.2	15.8	0.185	EUR	Valdiosera et al., *PNAS* (2018)[10]
Stuttgart	Germany	7020-7260	16.2	19.1	0.038*	EUR	Lazaridis et al., *Nature* (2014)[11]
Loschbour	Luxembourg	7940-8160	18.2	21.2	0.027*	EUR	Lazaridis et al., *Nature* (2014)[11]
Ballynahatty	Ireland	4970-5293	10.0	10.8	0.151	EUR	Cassidy et al., *PNAS* (2016)[12]
sf12	Sweden	8757-9033	58.9	74.4	UDG*	EUR	Günther et al., *PloS Biology* (2018)[13]
NE1	Hungary	7021-7256	18.4	21.8	0.096	EUR	Gamba et al., *Nat. Com.* (2014)[14]
RISE1159	Poland	4726-4830	27.5	33.5	0.110	EUR	Schroeder et al., *PNAS* (2019)[5]; Allentoft et al., *bioRxiv* (2022)[6]
RISE1160	Poland	4587-4841	5.4	7.1	0.166	-	Schroeder et al., *PNAS* (2019)[5]; Allentoft et al., *bioRxiv* (2022)[6]
RISE1168	Poland	4726-4830	18.9	23.4	0.112	EUR	Schroeder et al., *PNAS* (2019)[5]; Allentoft et al., *bioRxiv* (2022)[6]
SIII	Russia	33031-35154	11.2	11.9	0.016	EUR	Sikora et al., *Science* (2017)[4]
Rathlin1	Ireland	3835-3976	10.8	11.8	0.143	EUR	Cassidy et al., *PNAS* (2016)[12]
SSG-A-2	Iceland	950-1100	10.2	11.5	-	EUR	Ebenesersdottir et al., *Science* (2018)[15]
HSJ-A-1	Iceland	950-1080	29.4	33.3	-	EUR	Ebenesersdottir et al., *Science* (2018)[15]
STT-A-2	Iceland	950-1050	14.1	16.1	-	EUR	Ebenesersdottir et al., *Science* (2018)[15]
VK1	Greenland	750-950	12.1	14.4	0.096	EUR	Margaryan et al., *Nature* (2020)[16]
BR2	Hungary	3060-3220	18.3	21.7	0.073	EUR	Gamba et al., *Nat. Com.* (2014)[14]
SZ15	Hungary	1346-1538	10.6	11.6	UDG*	EUR	Amorim et al., *Nat. Com.* (2018)[17]
SZ3	Hungary	1346-1538	10.6	11.3	UDG*	EUR	Amorim et al., *Nat. Com.* (2018)[17]
SZ4	Hungary	1347-1538	10.4	11.1	UDG*	EUR	Amorim et al., *Nat. Com.* (2018)[17]
SZ45	Hungary	1347-1538	10.4	11.3	UDG*	EUR	Amorim et al., *Nat. Com.* (2018)[17]
SZ43	Hungary	1347-1538	11.6	12.5	UDG*	EUR	Amorim et al., *Nat. Com.* (2018)[17]
SZ1	Hungary	3220-5320	10.9	11.5	UDG*	EUR	Amorim et al., *Nat. Com.* (2018)[17]
baa01	South Africa	1831-1986	13.5	17.9	0.045	AFR	Schlebusch et al., *Science* (2017)[18]
ela01	South Africa	453-533	13.5	17.3	0.030	AFR	Schlebusch et al., *Science* (2017)[18]
new01	South Africa	327-508	10.9	14.4	0.037	AFR	Schlebusch et al., *Science* (2017)[18]
I10871	Cameroon	7800-7970	15.2	24.0	0.065	AFR	Lipson et al., *Nature* (2020)[19]
Mota	Ethiopia	4419-4525	10.2	11.5	0.210*	AFR	Gallego Llorente et al., *Science* (2015)[20]
KK1	Georgia	9550-9890	11.8	12.7	0.250*	EUR	Jones et al., *Nat. Com.*, (2015)[21]
WC1	Iran	9032-9405	10.4	11.9	0.220	EUR	Broushaki et al., *Science* (2016)[7]
BOT2016	Kazakhstan	5318-5582	13.9	15.0	0.208	EUR	Damgaard et al., *Science* (2018)[22]
Yamnaya	Kazakhstan	4837-4968	26.4	28.1	0.188	EUR	Damgaard et al., *Science* (2018)[22]
Andaman	India	30-150	16.6	19.0	0.049	SAS	Moreno-Mayar et al., *Science* (2018)[3]
UstIshim	Russia	42560-47480	35.2	41.6	0.022	All	Fu et al., *Nature* (2014)[23]
Yana	Russia	30950-32950	26.5	28.9	0.139	All	Sikora et al., *Nature* (2019)[24]
Kolyma_River	Russia	9665-9906	15.0	16.1	0.120	All	Sikora et al., *Nature* (2019)[24]
USR1	USA	11270-11600	17.1	18.6	0.011	AME	Moreno-Mayar et al., *Nature* (2018)[25]
AHUR_2064	USA	10770-11170	18.9	20.4	0.012	AME	Moreno-Mayar et al., *Science* (2018)[3]
Lovelock2	USA	1818-1942	15.4	16.6	0.008	AME	Moreno-Mayar et al., *Science* (2018)[3]
Lovelock3	USA	567-687	19.1	20.6	0.008	AME	Moreno-Mayar et al., *Science* (2018)[3]
Saqqaq	Greenland	3600-4170	13.4	17.1	0.008	AME	Rasmussen et al., *Nature* (2010)[26]
Clovis	USA	12572-12726	15.1	17.9	0.162	AME	Moreno-Mayar et al., *Science* (2018)[3]
Sumidouro5	Brazil	10258-10552	15.8	16.8	0.420	AME	Moreno-Mayar et al., *Science* (2018)[3]
A460	Chile	4430-4850	11.1	11.9	0.150	AME	Moreno-Mayar et al., *Science* (2018)[3]

Supplementary Table 2. Sample categories defined in this study

ID	Sample category
atp016	Prehistoric Europe
Stuttgart	Prehistoric Europe
Loschbour	Prehistoric Europe
Ballynahatty	Prehistoric Europe
sf12	Prehistoric Europe
NE1	Prehistoric Europe
RISE1159	Prehistoric Europe
RISE1160	Prehistoric Europe
RISE1168	Prehistoric Europe
SIII	Prehistoric Europe
Rathlin1	Historic Europe
SSG-A-2	Historic Europe
HSJ-A-1	Historic Europe
STT-A-2	Historic Europe
VK1	Historic Europe
BR2	Historic Europe
SZ15	Historic Europe
SZ3	Historic Europe
SZ4	Historic Europe
SZ45	Historic Europe
SZ43	Historic Europe
SZ1	Historic Europe
baa01	Africa
ela01	Africa
new01	Africa
I10871	Africa
Mota	Africa
KK1	Western Asia
WC1	Western Asia
BOT2016	Western Asia
Yamnaya	Western Asia
Andaman	South Asia
UstIshim	Siberia
Yana	Siberia
Kolyma_River	Siberia
USR1	Americas
AHUR_2064	Americas
Lovelock2	Americas
Lovelock3	Americas
Saqqaq	Americas
Clovis	Americas
Sumidouro5	Americas
A460	Americas

Supplementary Note 4: Comparing imputation of ancient and present-day genomes

To determine how imputation of ancient and present-day genomes differ, we downsampled to 1x and imputed 23 present-day European high-coverage genomes present in the Simons Diversity Project (SGDP[27]) dataset (Supplementary Table 3). We subsequently assessed imputation performance for these genomes and compared with the results obtained when imputing 21 ancient European genomes (nine and 12 in the "Prehistoric Europe" and "Historic Europe" categories, respectively), which had been previously downsampled to 1x.

We found that the imputation accuracy of the abovementioned present-day genomes was similar to what we had obtained in the case of the European ancient genomes (Supplementary Fig. 3). At SNPs with MAF between 0.1% and 1%, r^2 (imputation accuracy) was 0.78, 0.85 and 0.81 for the prehistoric, ancient historic and present-day European genomes, respectively (Supplementary Fig. 3a). For sites with MAF>1%, the r^2 values of the three datasets converged, with a minimum value of around 0.90. The same trend can be seen when comparing non-reference discordance (NRD) values per sample (Supplementary Fig. 3b). The mean NRD was 2.80%, 2.21% and 2.78% and was in the range 2.05%-3.57%, 1.29%-3.10% and 2.01%-3.99% for the prehistoric, ancient historic and present-day European genomes, respectively.

Supplementary Table 3. Subset of 23 European SGDP genomes used to compare imputation of ancient and present-day genomes. In this table, we report the SGDP sample IDs, the countries of origin and mean depths of coverage, as reported in Mallick et al[27].

ID (SGDP)	Country	Coverage
S_Albanian-1	Albania	36.2
S_Bulgarian-1	Bulgaria	42.7
S_Bulgarian-2	Bulgaria	37.5
S_Czech-2	Czechoslovia(pre1989)	49.7
S_Estonian-2	Estonia	35.2
S_Estonian-1	Estonia	38.4
S_French-1	France	44.3
S_French-2	France	40.2
S_Basque-1	France	41.9
S_Basque-2	France	38.5
B_French-3	France	43.4
S_Greek-1	Greece	37.7
S_Greek-2	Greece	45.5
B_Crete-2	Greece	42.7
S_Hungarian-2	Hungary	40.7
S_Hungarian-1	Hungary	40.1
S_Icelandic-1	Iceland	38.3
S_Icelandic-2	Iceland	49.3
B_Sardinian-3	Italy	39.5
S_Bergamo-1	Italy(Bergamo)	68.6
S_Bergamo-2	Italy(Bergamo)	80.0
S_Sardinian-1	Italy(Sardinia)	36.4
S_Sardinian-2	Italy(Sardinia)	34.8

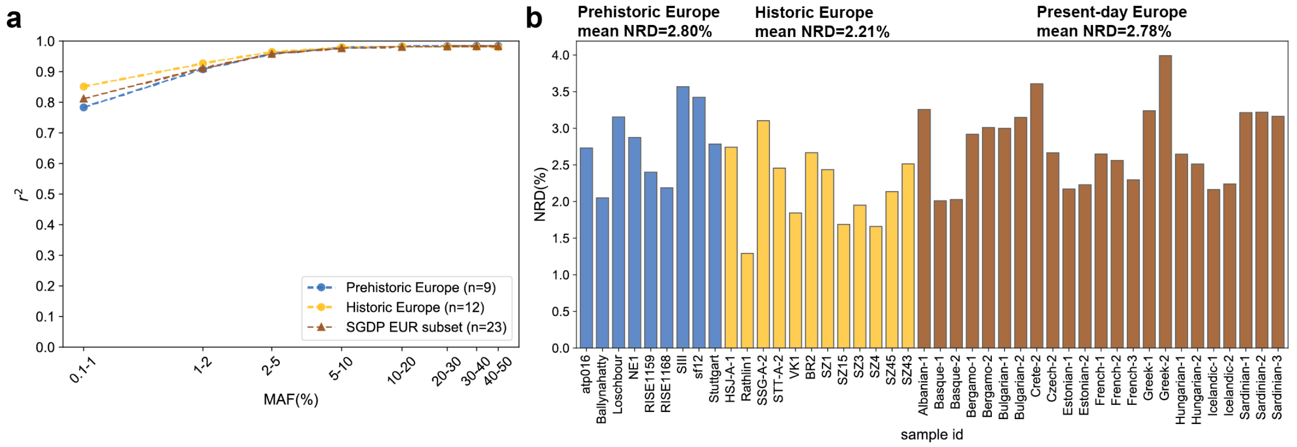

Supplementary Fig. 3. Imputation performance of ancient and present-day 1x European genomes. a Imputation accuracy, r^2, as a function of minor allele frequency, MAF, for the aggregate of prehistoric (blue), ancient historic Europeans (yellow) and a subset of 23 present-day individuals in SGDP (brown). **b** Non-reference discordance (NRD) per individual sample.

Supplementary Note 5: Imputation accuracy for transitions and transversions and method comparison

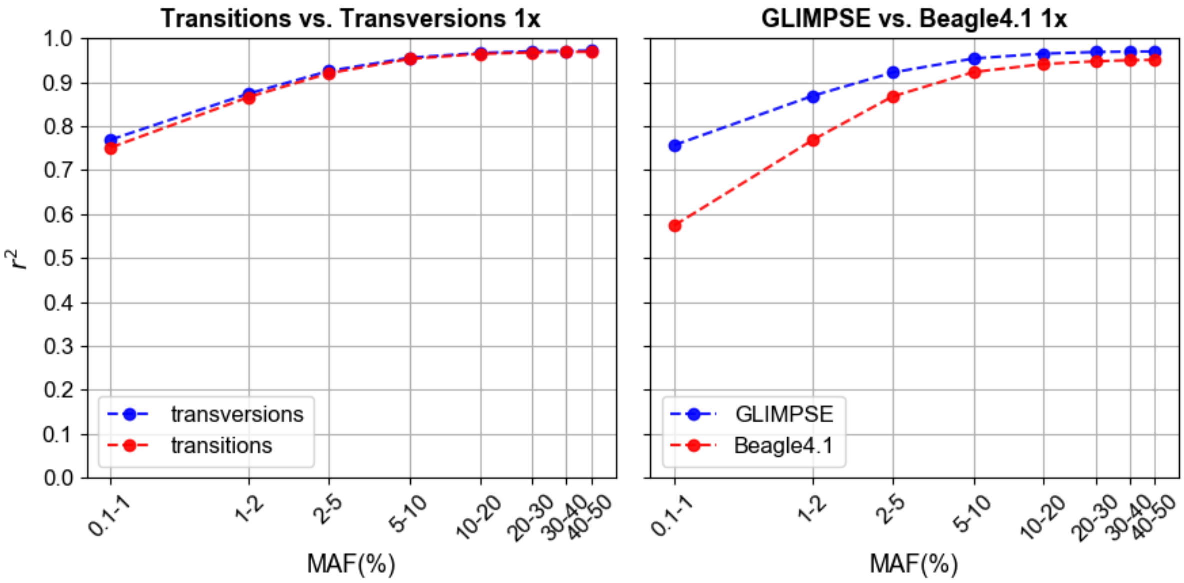

Supplementary Fig. 4. Comparing imputation performance between mutation types and imputation methos. Imputation accuracy, r^2, for the aggregate of the 42 ancient genomes, previously downsampled to 1x, as a function of 1000 Genomes Project minor allele frequency (MAF), 0.1-50%, regarding (left to right) i) transitions (green) compared to transversions (purple), and ii) comparison between imputation methods, Beagle4.1[28] (green) vs. GLIMPSE1.1.1[29] (purple).

Supplementary Note 6: Genotype discordance between imputed 1x and high-coverage genomes

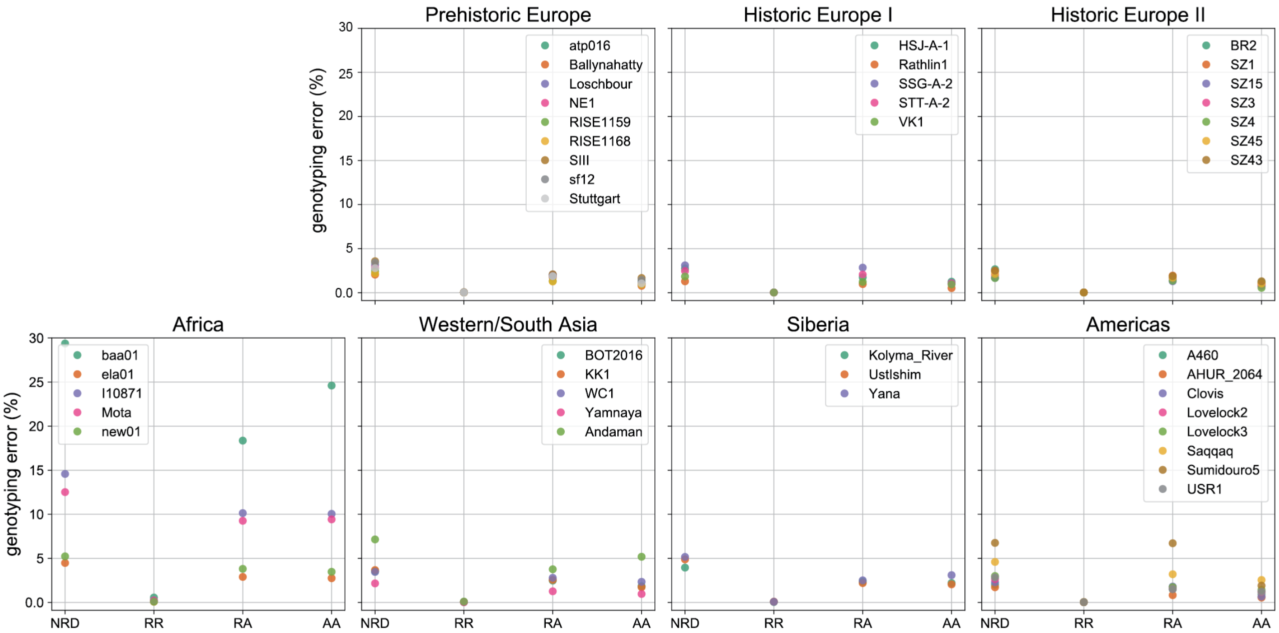

Supplementary Fig. 5. Imputation genotyping errors for 1x genomes. Genotype discordance between individual imputed (1x) and high-coverage genomes for homozygous reference allele (RR), heterozygous (RA) and homozygous alternative allele (AA) sites, as well as the resulting non-reference discordance (NRD).

Supplementary Note 7: Imputing ancient Native American genomes using subsets of the 1000 Genomes reference panel

The ancient Native American genomes were accurately imputed, even though these ancestries are only represented through admixed individuals in the American reference populations. To understand whether it was these populations' presence in the 1000 Genomes reference panel that allowed for such high imputation accuracy, we further imputed the eight Native American ancient genomes (1x) using subsets of the 1000 Genomes panel. We generated five different subsets by removing one continental group (of size around 500) for each so that the subset sizes would be approximately the same. Comparing to imputation with the full reference panel, imputation accuracy remained unaltered when removing a continental group, except when removing the 1000G American

populations (Supplementary Fig. 6). This hints that GLIMPSE is only copying from American haplotypes in the reference panel. Without this reference population, imputation accuracy drastically dropped at rare variants (MAF<2%), reaching 0.46 at MAF between 0.1% and 1% (vs. 0.78 in the remaining five imputation experiments). The difference in imputation accuracy was much smaller at common variants, where the values converged across the different experiments as MAF increased. This analysis shows that the American populations in 1000G greatly contributed to imputation of rare variants in the Native Americans ancient individuals. Nonetheless, common variants (MAF>5%) were accurately imputed even when the American reference populations were removed. This suggests that poorly represented non-African genomes can still be accurately imputed when restricting to common variants, likely due to a more recent common history, that is, smaller coalescent distance, between non-Africans.

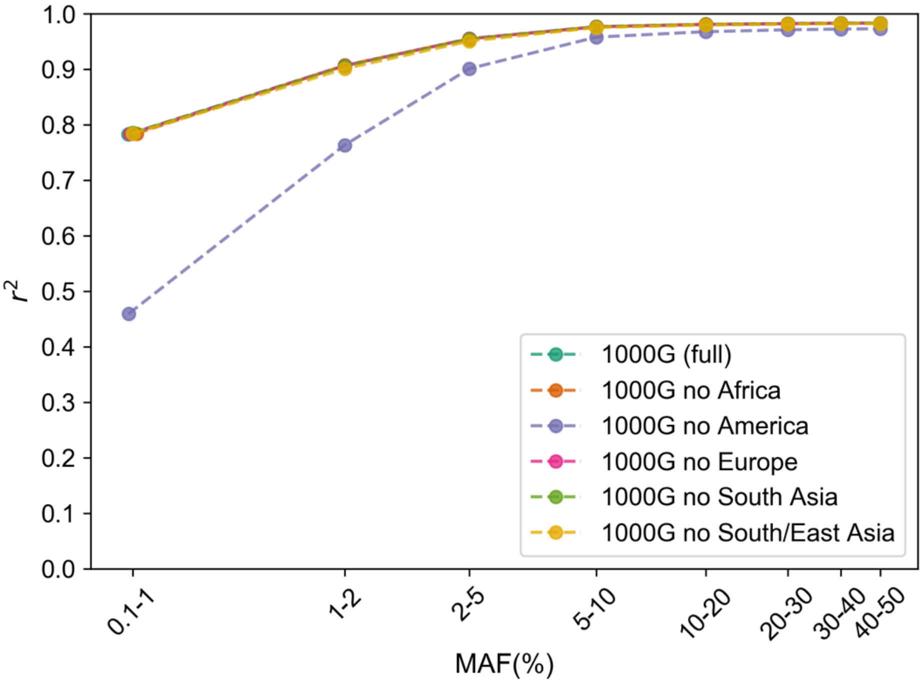

Supplementary Fig. 6. Contribution of 1000 Genomes continental groups to imputation of ancient Native American genomes. Imputation accuracy, r^2, as a function of minor allele frequency (MAF) of the aggregate of eight 1x ancient Native Americans genomes when using the full 1000 Genomes as a reference panel and when removing one continental group at a time.

Supplementary Note 8: Effect of sample age on imputation accuracy

To verify whether sample age impacts imputation performance, we plotted imputation accuracy (r^2) against sample age for all the 42 imputed 1x ancient data for which we have available high-coverage genomes. Given the observation that imputation accuracy is much lower at rare variants (between 0.4 and 0.6 when MAF is between 0.1% and 1%) for the oldest samples in our dataset (Figure 2a), i.e., SIII (~34,000 ybp), Yana (~32,000 ybp) and Ust'Ishim (~45,000 ybp), we split this analysis in the eight MAF bins for which we calculated imputation accuracy (Supplementary Fig. 7). For each MAF bin, we computed Spearman correlation (r_S) between age and r^2. We indeed found significantly negative correlation values (at 5% threshold) for the lowest MAF bins, i.e., 0.1%-1% and 1%-2%: r_s=-0.465 (p-value=0.002) and r_s=-0.324 (p-value=0.036), respectively. For more common variants, no relationship between age and imputation accuracy emerged.

Here we showed that age negatively affects imputation accuracy at rare variants. However, at such variants, the imputation accuracies of the three oldest samples and the youngest sample (Andaman) are similar. The Andaman genome comes from an isolated population that is not well represented in the 1000 Genomes reference panel. This observation hints that a large distance in time translates into genetic differences to the reference panel in a similar way as a more recent, but more distant to the reference panel non-African genome.

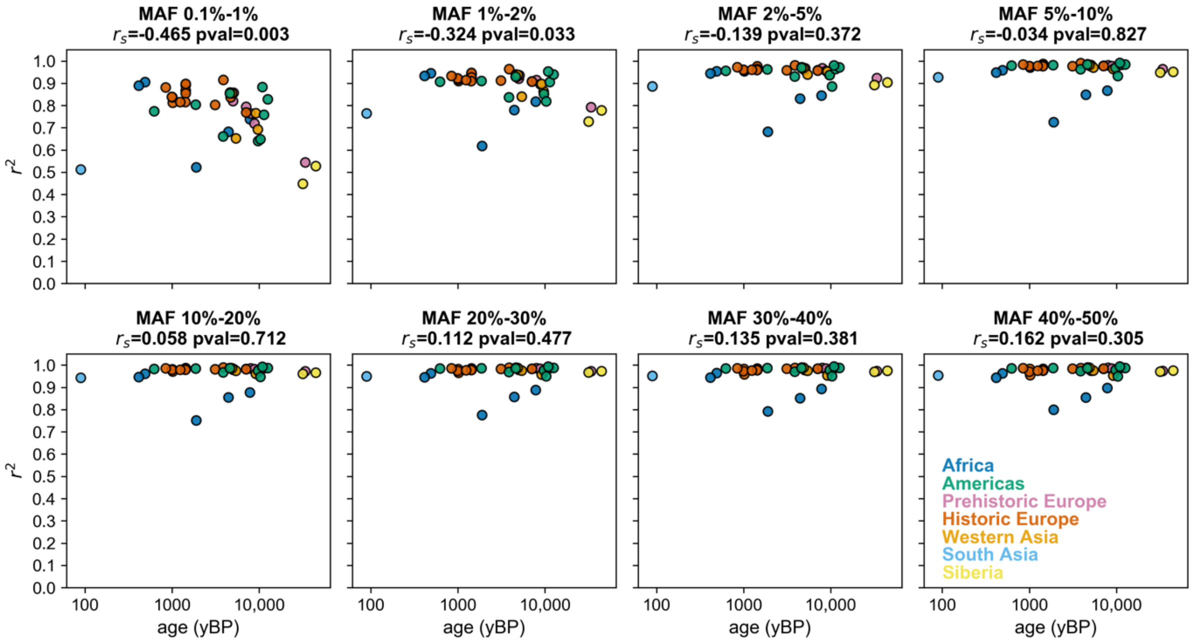

Supplementary Fig. 7. Effect of sample age on imputation. Relationship between sample age (*x*-axis) on imputation accuracy, r^2 (*y*-axis), split in eight minor allele frequency (MAF) bins. For each MAF bin, we report the Spearman correlation (r_s) between r^2 and age, and the corresponding *p*-value, that resulted from testing whether the two variables were significantly correlated using a two-sided permutation test with 10,000 permutations (n=42). No adjustments were made for multiple comparisons. Each dot corresponds to an imputed 1x genome.

Supplementary Note 9: Imputation of ancient capture data

Supplementary Table 4. Individual samples for which we had access to both whole-genome sequencing (WGS) and capture (on the 1240K array) data. Here, we report the depth of coverage of the capture data at the intersection of the 1240K and 1000 Genomes panel variant sites and at variants contained in the 1000 Genomes panel alone. We also report the depth of coverage of the corresponding WGS data.

Sample Id	Capture coverage (1240K in 1000G)	Capture coverage (all sites 1000G)	Shotgun-sequenced genome coverage (all sites 1000G)
BOT2016	2.6	0.2	15.0
BR2	1.5	0.1	21.7
I10871	8.5	0.9	24.0
NE1	1.7	0.2	21.8
Stuttgart	11.1	0.7	19.1

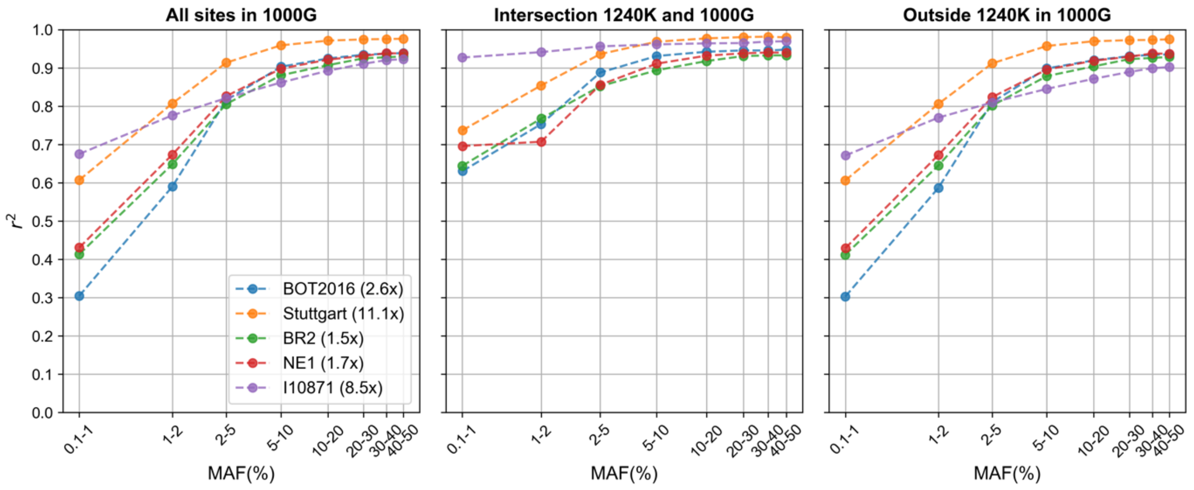

Supplementary Fig. 8. Imputation of ancient capture genomes. Imputation accuracy, r^2, as a function of minor allele frequency (MAF) when imputing the five capture genomes at full coverage at, from left to right, all variant sites in 1000 Genomes umich reference panel, the sites in the intersection of the latter with 1240K dataset, and the sites in 1000 Genomes umich outside of 1240K.

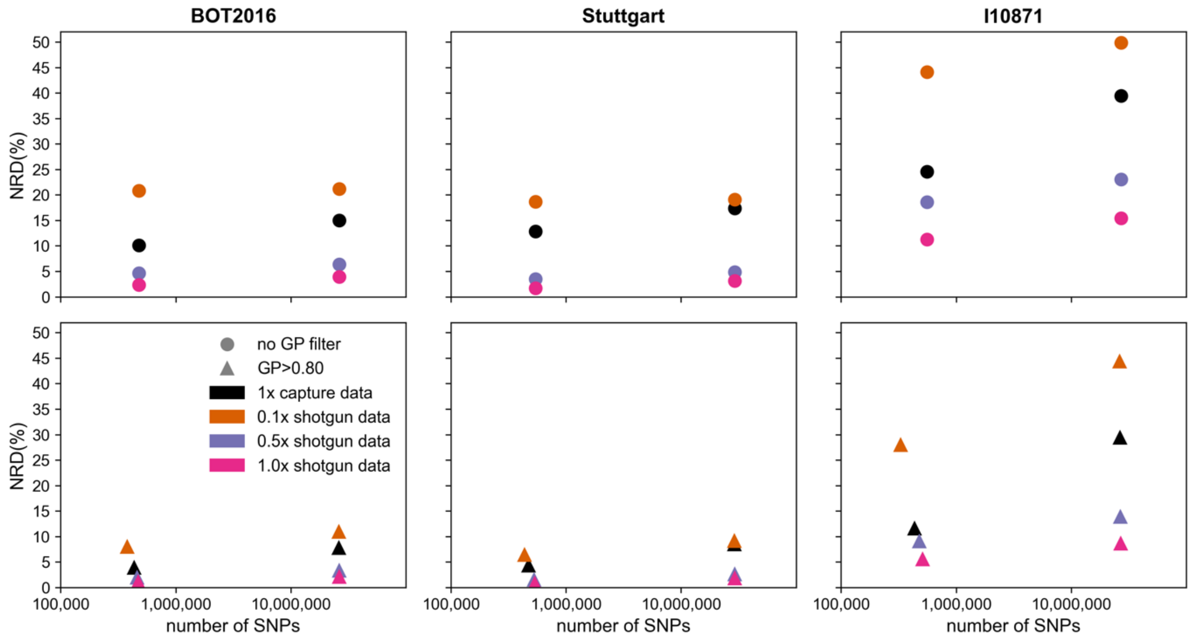

Supplementary Fig. 9. Non-reference discordance (NRD) values against the number of SNPs obtained when imputing 1.0x capture genomes and whole genome data (0.1x, 0.5x and 1.0x) for the same individual samples (BOT2016, Stuttgart and I10871). We report NRD at both the intersection of 1000G and 1240K sites and the 1000G sites located outside of this intersection. Top: no genotype probability (GP) filter; bottom: GP>0.80.

Supplementary Note 10: PCA with focus on Europeans, several coverages

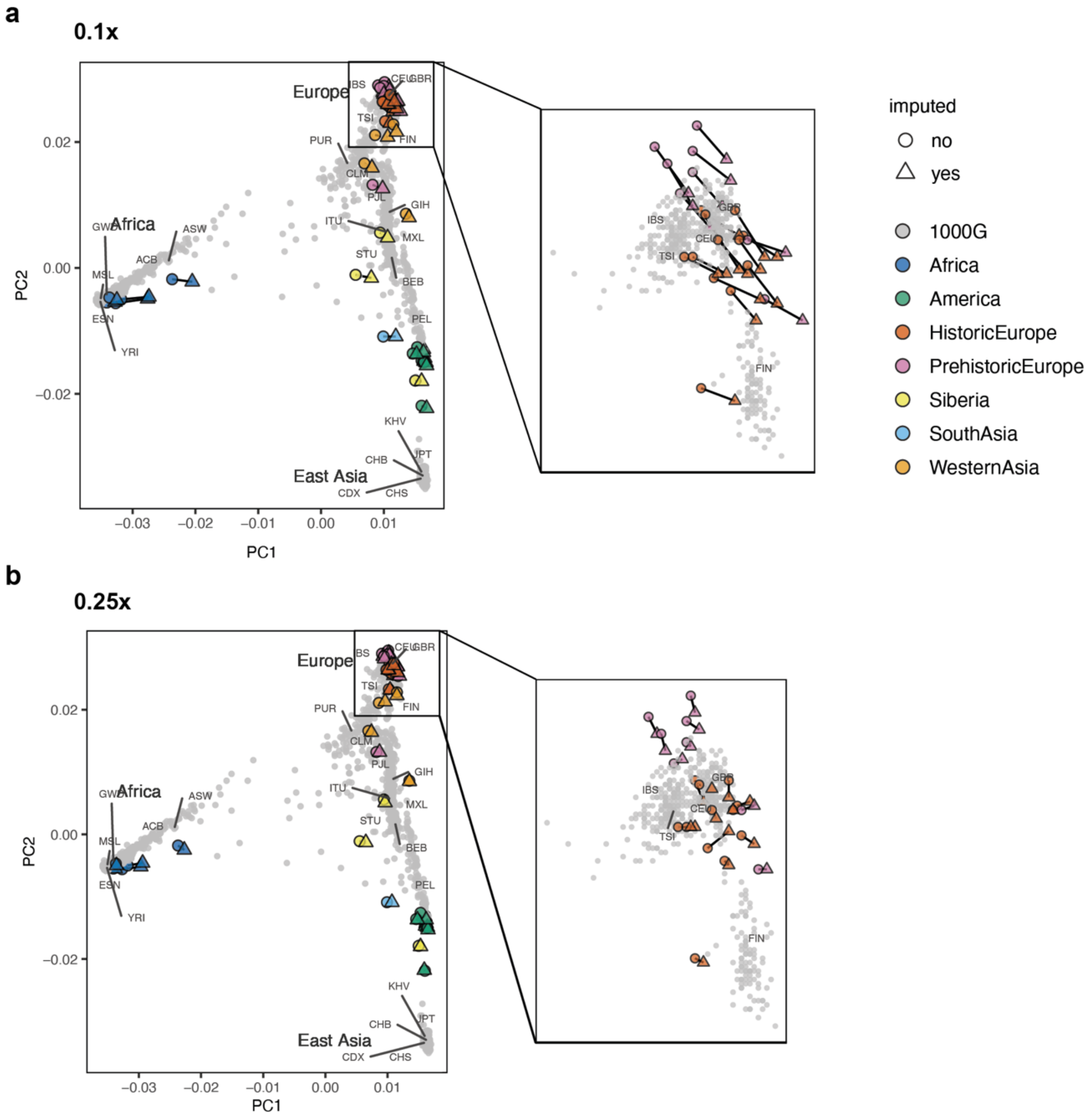

Supplementary Fig. 10. Effect of imputing lower coverage ancient genomes on principal component analysis (PCA). Principal component analysis (PCA) of imputed **a** 0.1x and **b** 0.25x and high-coverage ancient genetic data, and present-day data in 1000 Genomes reference panel (gray). Plots show individual coordinates along the two first principal components, zooming-in on the individuals of European ancestry. Imputed data points are represented by triangles and high-coverage ancient data by full circles. Corresponding imputed and high-coverage data are connected by a line.

Supplementary Note 11: Genetic clustering analyses: from K=2 to K=5 clustering populations

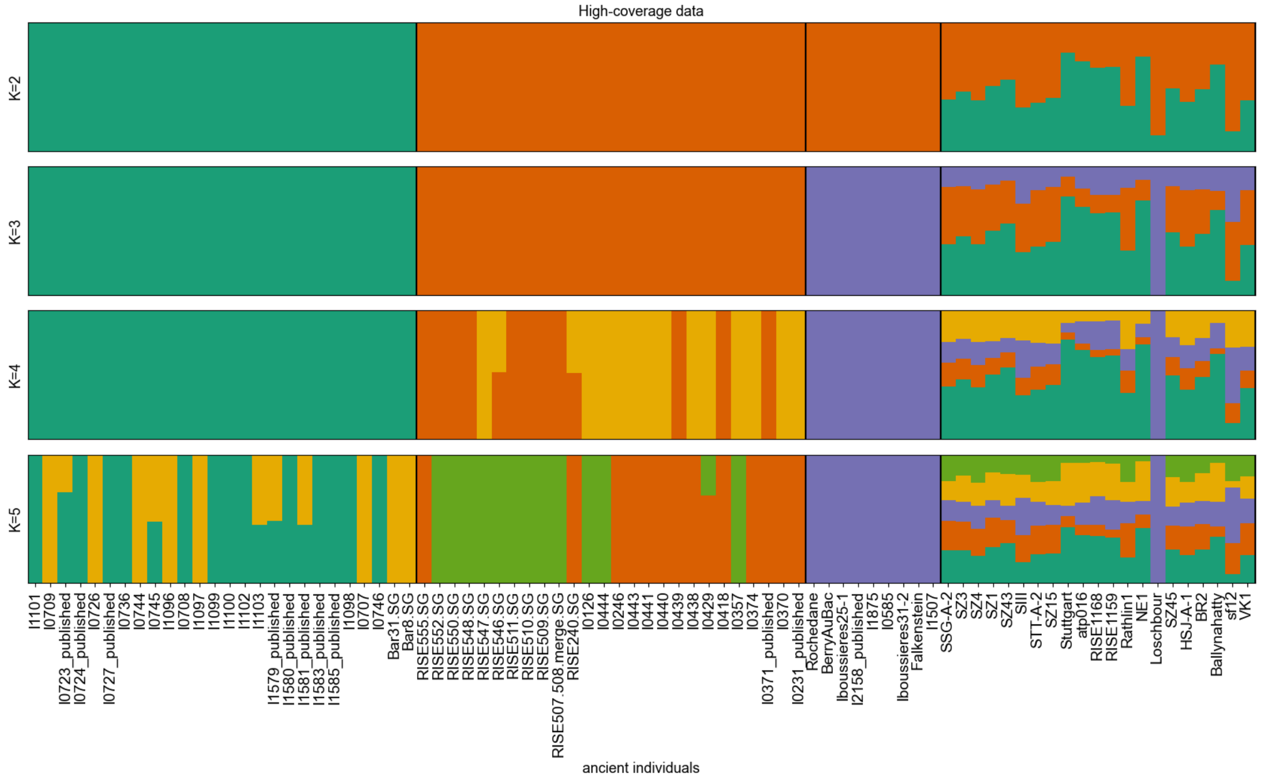

Supplementary Fig. 11. Genetic clustering estimates for 21 high-coverage European ancient genomes. Genetic clustering results obtained from running unsupervised ADMIXTURE[30] with the genetic data of a subset of individuals in 1240K dataset[31] and high-coverage data (rightmost box), with varying number of clustering populations (K between 2 and 5).

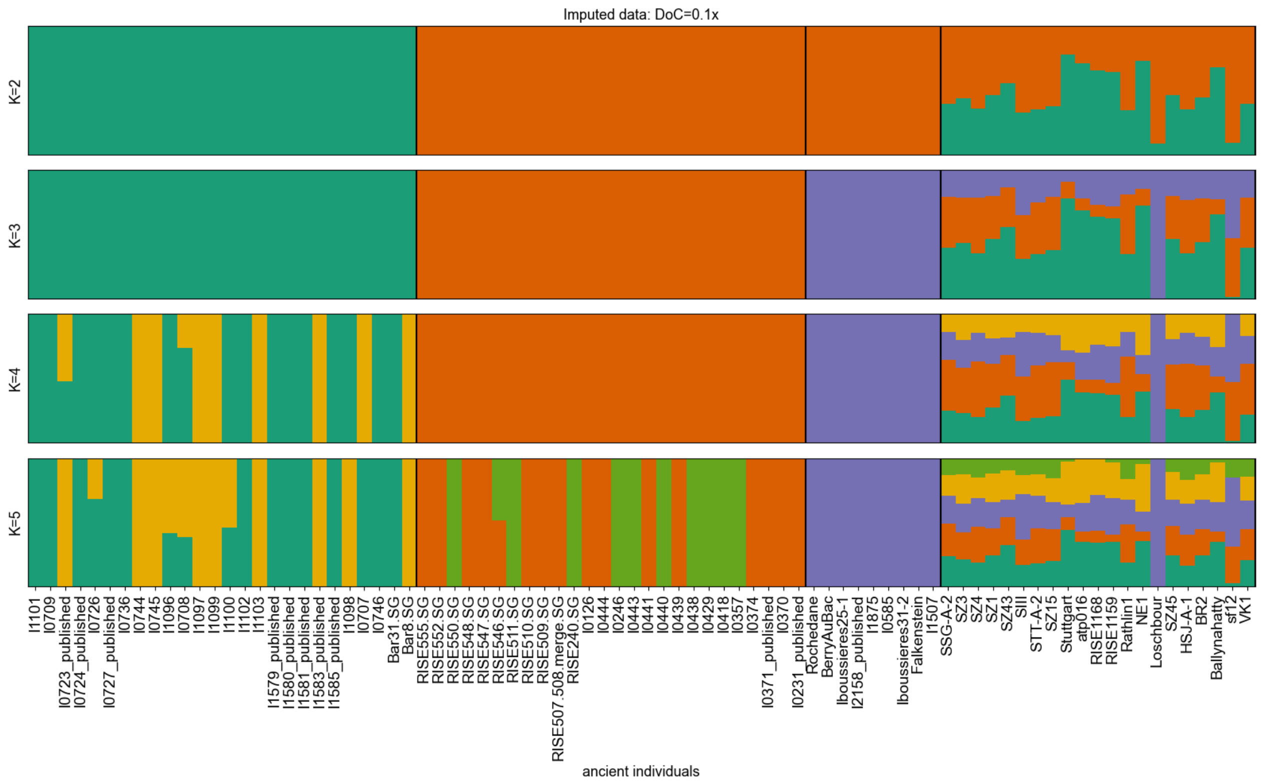

Supplementary Fig. 12. Genetic clustering estimates for 21 0.1x imputed European ancient genomes. Genetic clustering results obtained from running unsupervised ADMIXTURE with the genetic data of a subset of individuals in 1240K dataset and imputed 0.1x data (rightmost box), with varying number of clustering populations (K between 2 and 5).

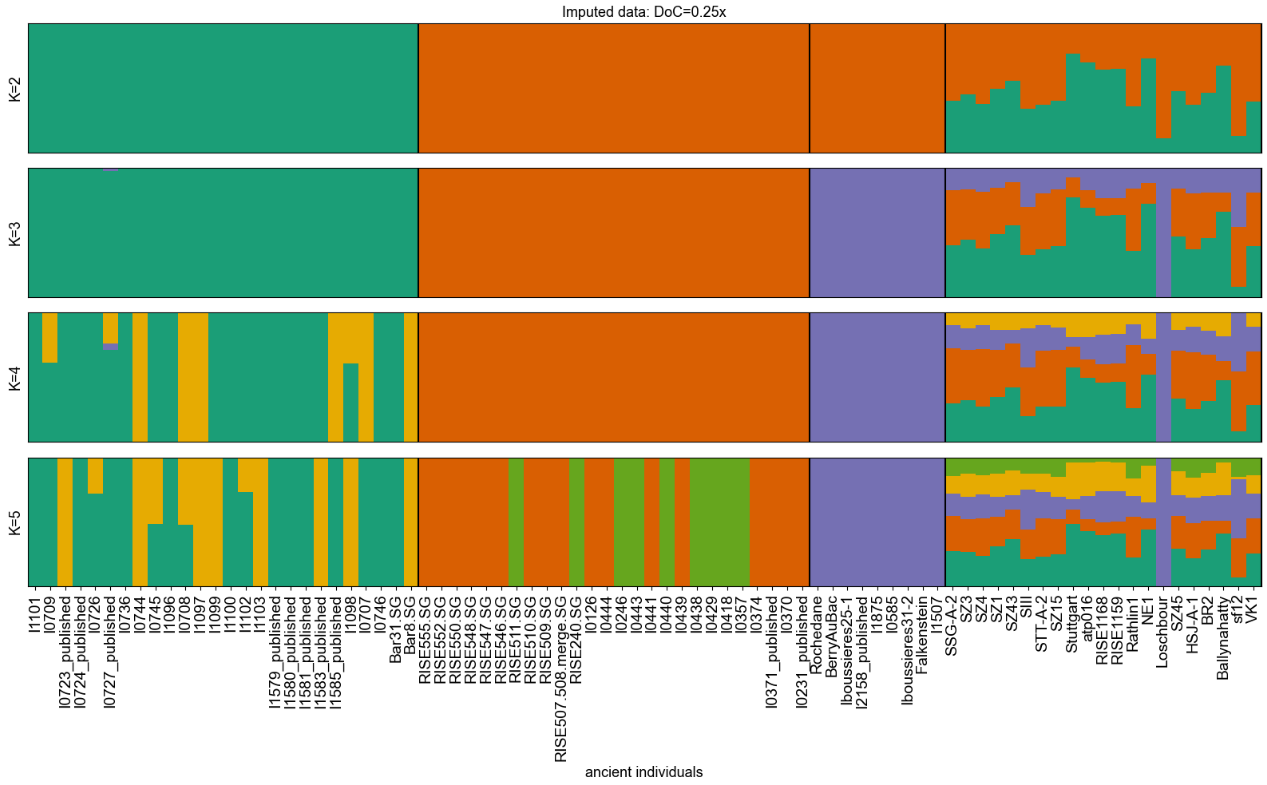

Supplementary Fig. 13. Genetic clustering estimates for 21 0.25x imputed European ancient genomes. Genetic clustering results obtained from running unsupervised ADMIXTURE with the genetic data of a subset of individuals in 1240K dataset and imputed 0.25x data (rightmost box), with varying number of clustering populations (K between 2 and 5).

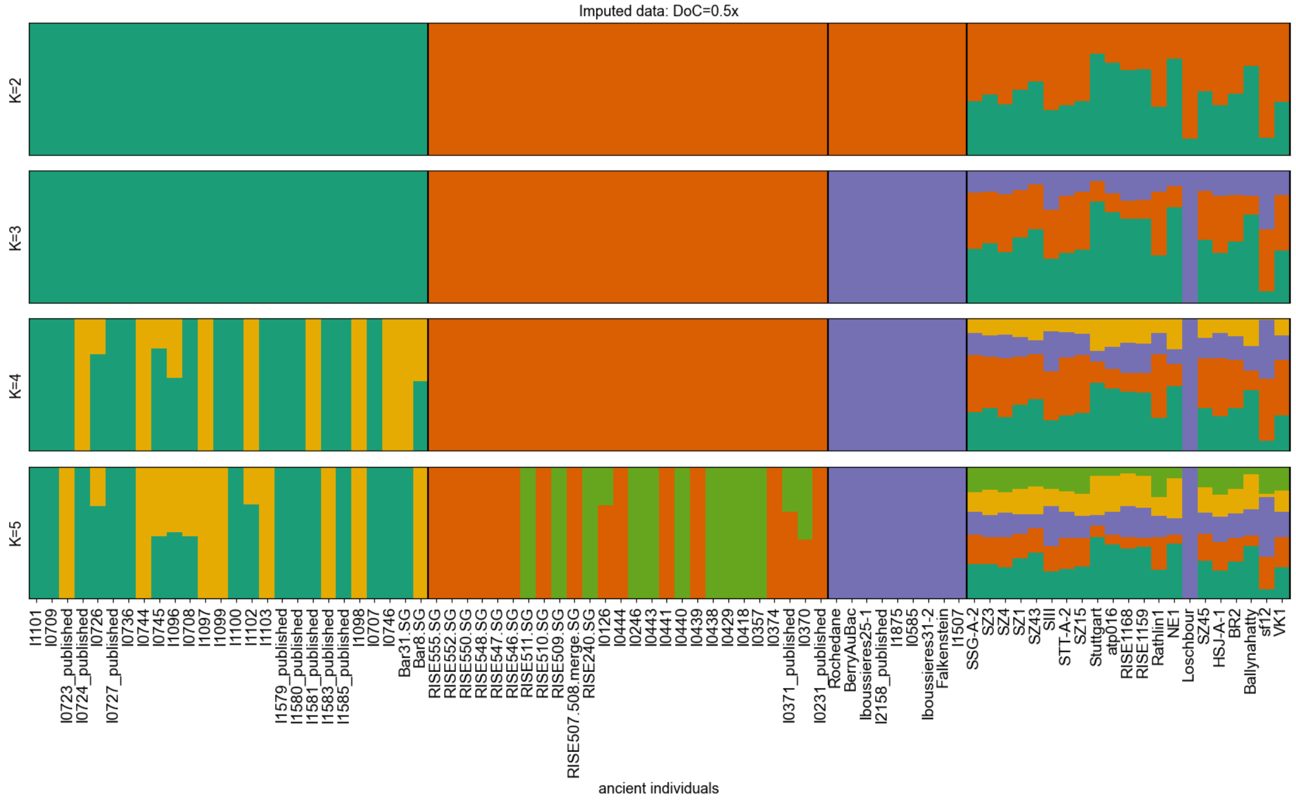

Supplementary Fig. 14. Genetic clustering estimates for 21 0.5x imputed European ancient genomes. Genetic clustering results obtained from running unsupervised ADMIXTURE with the genetic data of a subset of individuals in 1240K dataset and imputed 0.5x data (rightmost box), with varying number of clustering populations (K between 2 and 5).

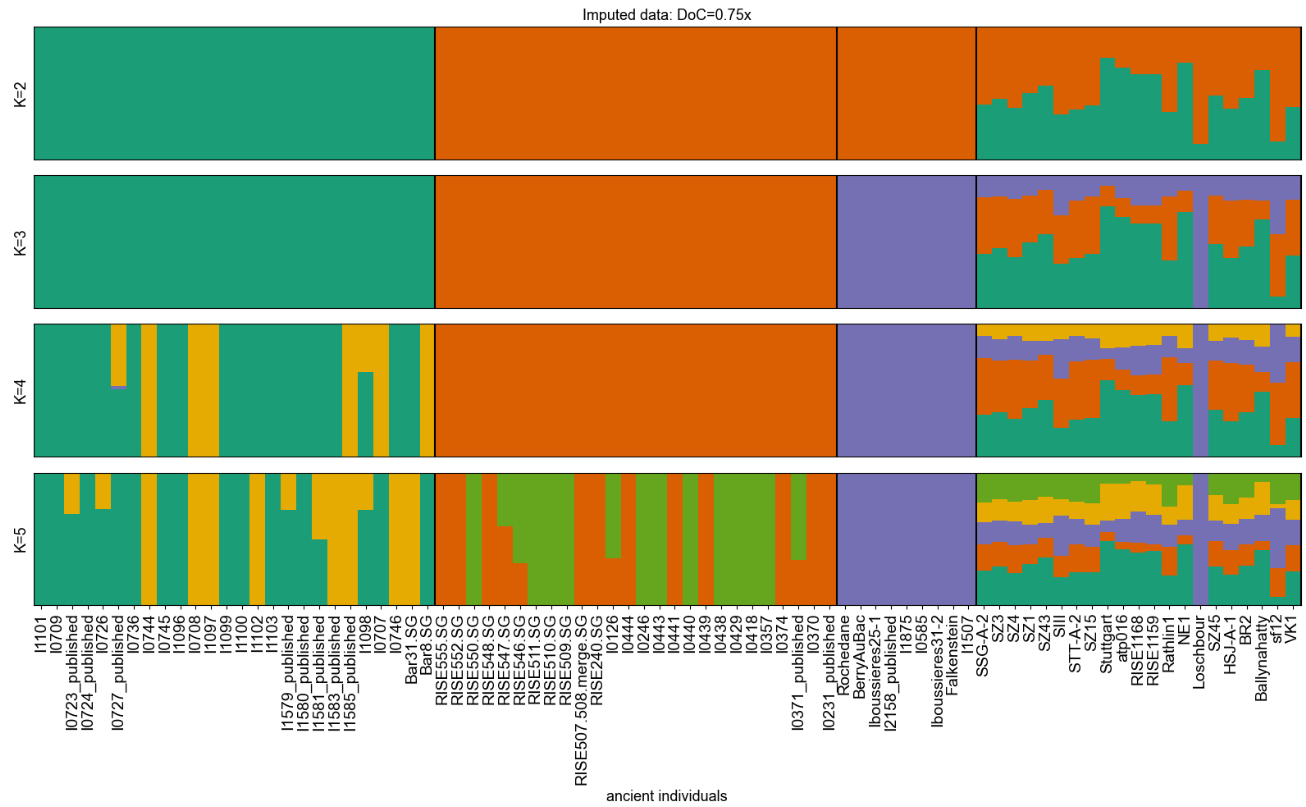

Supplementary Fig. 15. Genetic clustering estimates for 21 0.75x imputed European ancient genomes. Genetic clustering results obtained from running unsupervised ADMIXTURE with the genetic data of a subset of individuals in 1240K dataset and imputed 0.75x data (rightmost box), with varying number of clustering populations (K between 2 and 5).

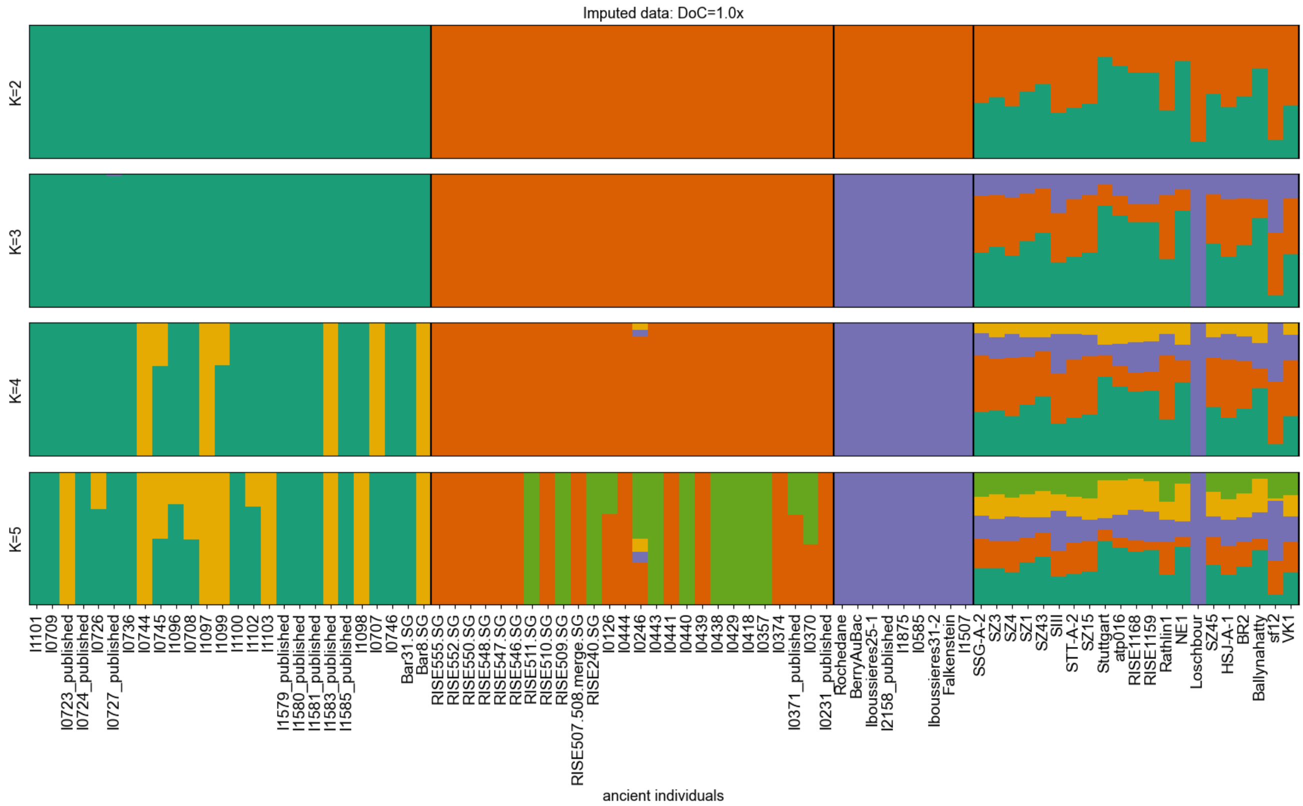

Supplementary Fig. 16. Genetic clustering estimates for 21 1.0x imputed European ancient genomes. Genetic clustering results obtained from running unsupervised ADMIXTURE with the genetic data of a subset of individuals in 1240K dataset and imputed 1.0x data (rightmost box), with varying number of clustering populations (K between 2 and 5).

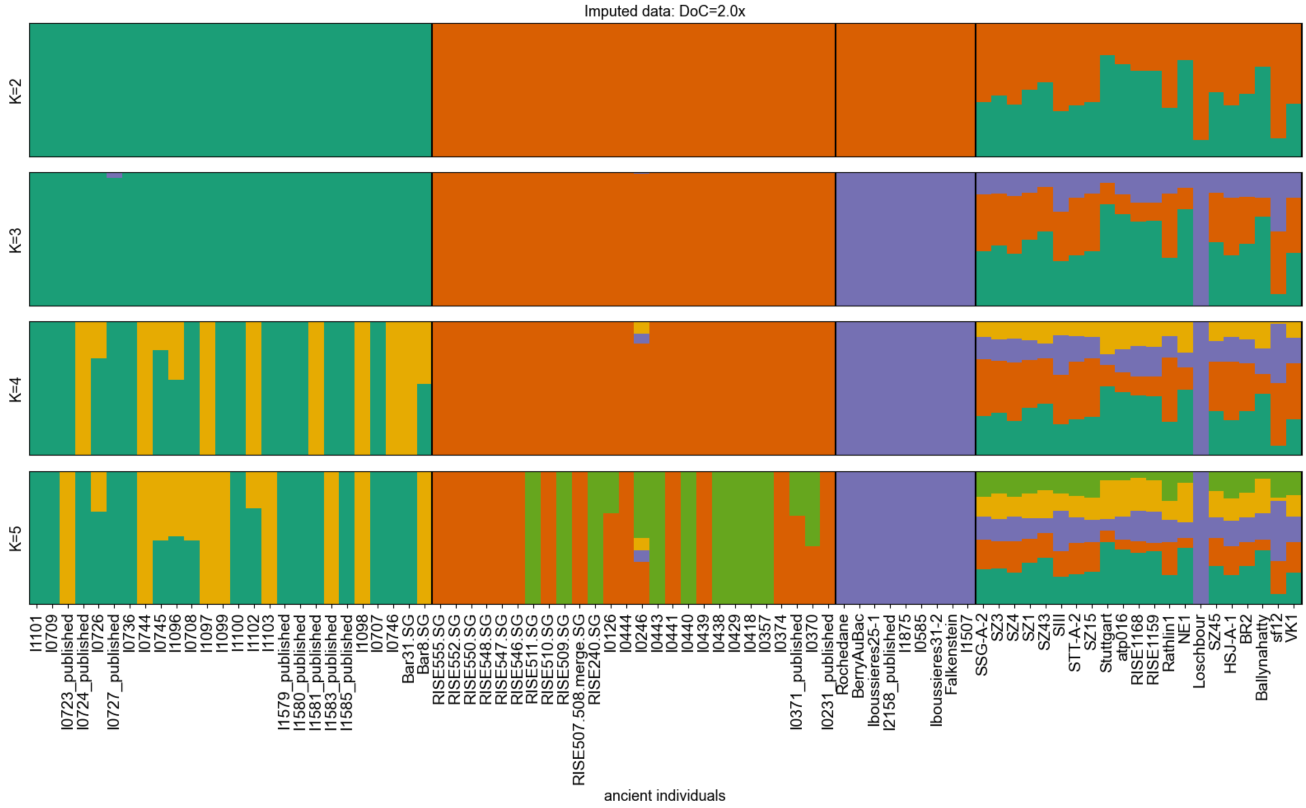

Supplementary Fig. 17. Genetic clustering estimates for 21 2.0x imputed European ancient genomes. Genetic clustering results obtained from running unsupervised ADMIXTURE with the genetic data of a subset of individuals in 1240K dataset and imputed 2.0x data (rightmost box), with varying number of clustering populations (K between 2 and 5).

Supplementary Table 5. Subset of the 1240K dataset[32] used as reference in the genetic clustering analyses. The individuals are grouped by population: Neolithic Anatolia (“Anatolia_N”), western hunter-gatherer (“WHG”), Early to Middle Bronze Age Steppe (“Steppe_EMBA”).

Population	Individual	Publication
Anatolia_N	Bar31.SG	Hofmanová et *al.*, *PNAS* (2016)[33]
Anatolia_N	Bar8.SG	Hofmanová et *al.*, *PNAS* (2016)[33]
Anatolia_N	I0707	Mathieson et *al.*, *Nature* (2015)[34]
Anatolia_N	I0708	Mathieson et *al.*, *Nature* (2015)[34]
Anatolia_N	I0709	Mathieson et *al.*, *Nature* (2015)[34]
Anatolia_N	I0723_published	Mathieson et *al.*, *Nature* (2015)[34]
Anatolia_N	I0724_published	Mathieson et *al.*, *Nature* (2015)[34]
Anatolia_N	I0726	Mathieson et *al.*, *Nature* (2015)[34]
Anatolia_N	I0727_published	Mathieson et *al.*, *Nature* (2015)[34]
Anatolia_N	I0736	Mathieson et *al.*, *Nature* (2015)[34]
Anatolia_N	I0744	Mathieson et *al.*, *Nature* (2015)[34]
Anatolia_N	I0745	Mathieson et *al.*, *Nature* (2015)[34]
Anatolia_N	I0746	Mathieson et *al.*, *Nature* (2015)[34]
Anatolia_N	I1096	Mathieson et *al.*, *Nature* (2015)[34]
Anatolia_N	I1097	Mathieson et *al.*, *Nature* (2015)[34]
Anatolia_N	I1098	Mathieson et *al.*, *Nature* (2015)[34]
Anatolia_N	I1099	Mathieson et *al.*, *Nature* (2015)[34]
Anatolia_N	I1100	Mathieson et *al.*, *Nature* (2015)[34]
Anatolia_N	I1101	Mathieson et *al.*, *Nature* (2015)[34]
Anatolia_N	I1102	Mathieson et *al.*, *Nature* (2015)[34]
Anatolia_N	I1103	Mathieson et *al.*, *Nature* (2015)[34]
Anatolia_N	I1579_published	Mathieson et *al.*, *Nature* (2015)[34]
Anatolia_N	I1580_published	Mathieson et *al.*, *Nature* (2015)[34]
Anatolia_N	I1581_published	Mathieson et *al.*, *Nature* (2015)[34]
Anatolia_N	I1583_published	Mathieson et *al.*, *Nature* (2015)[34]
Anatolia_N	I1585_published	Mathieson et *al.*, *Nature* (2015)[34]
WHG	BerryAuBac	Mathieson et *al.*, *Nature* (2018)[35]
WHG	Falkenstein	Mathieson et *al.*, *Nature* (2018)[35]
WHG	I0585	Mathieson et *al.*, *Nature* (2015)[34]
WHG	I1507	Mathieson et *al.*, *Nature* (2015)[34]
WHG	I1875	Mathieson et *al.*, *Nature* (2018)[35]
WHG	I2158_published	Mathieson et *al.*, *Nature* (2018)[35]
WHG	Iboussieres25-1	Mathieson et *al.*, *Nature* (2018)[35]
WHG	Iboussieres31-2	Mathieson et *al.*, *Nature* (2018)[35]
WHG	Rochedane	Mathieson et *al.*, *Nature* (2018)[35]
Steppe_EMBA	I0126	Mathieson et *al.*, *Nature* (2015)[34]
Steppe_EMBA	I0231_published	Narasimhan et *al.*, *Science* (2019)[36]
Steppe_EMBA	I0246	Narasimhan et *al.*, *Science* (2019)[36]
Steppe_EMBA	I0357	Mathieson et *al.*, *Nature* (2015)[34]
Steppe_EMBA	I0370	Mathieson et *al.*, *Nature* (2015)[34]
Steppe_EMBA	I0371_published	Mathieson et *al.*, *Nature* (2015)[34]
Steppe_EMBA	I0374	Mathieson et *al.*, *Nature* (2015)[34]
Steppe_EMBA	I0418	Mathieson et *al.*, *Nature* (2015)[34]
Steppe_EMBA	I0429	Mathieson et *al.*, *Nature* (2015)[34]
Steppe_EMBA	I0438	Mathieson et *al.*, *Nature* (2015)[34]
Steppe_EMBA	I0439	Mathieson et *al.*, *Nature* (2015)[34]
Steppe_EMBA	I0440	Mathieson et *al.*, *Nature* (2015)[34]
Steppe_EMBA	I0441	Mathieson et *al.*, *Nature* (2015)[34]
Steppe_EMBA	I0443	Mathieson et *al.*, *Nature* (2015)[34]
Steppe_EMBA	I0444	Mathieson et *al.*, *Nature* (2015)[34]
Steppe_EMBA	RISE240.SG	Allentoft et *al.*, *Nature* (2015)[37]
Steppe_EMBA	RISE507.508.merge.SG	Allentoft et *al.*, *Nature* (2015)[37]
Steppe_EMBA	RISE509.SG	Allentoft et *al.*, *Nature* (2015)[37]
Steppe_EMBA	RISE510.SG	Allentoft et *al.*, *Nature* (2015)[37]
Steppe_EMBA	RISE511.SG	Allentoft et *al.*, *Nature* (2015)[37]
Steppe_EMBA	RISE546.SG	Allentoft et *al.*, *Nature* (2015)[37]
Steppe_EMBA	RISE547.SG	Allentoft et *al.*, *Nature* (2015)[37]
Steppe_EMBA	RISE548.SG	Allentoft et *al.*, *Nature* (2015)[37]
Steppe_EMBA	RISE550.SG	Allentoft et *al.*, *Nature* (2015)[37]
Steppe_EMBA	RISE552.SG	Allentoft et *al.*, *Nature* (2015)[37]
Steppe_EMBA	RISE555.SG	Allentoft et *al.*, *Nature* (2015)[37]

Supplementary Note 12: ROH estimates for Sumidouro5

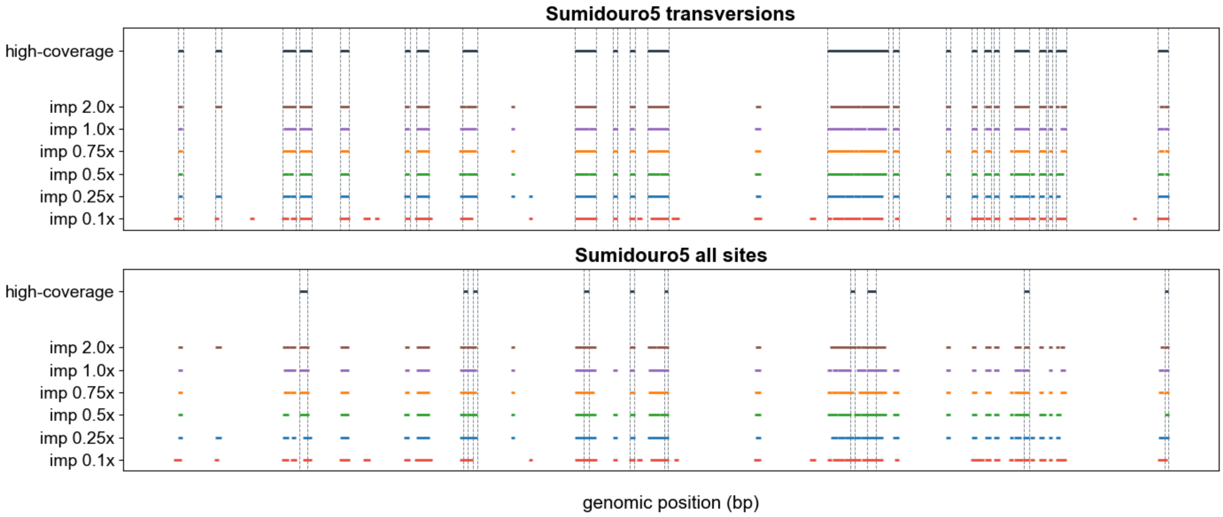

Supplementary Fig. 18. ROH in Sumidouro5: imputation seems to correct C-to-T mismatches. ROH segments identified in chromosome 10 for high-coverage and imputed data (DoC between 0.1x and 2.0x) for Sumidouro5. Top: ROH obtained using all sites. Bottom: ROH estimated using transversion sites only.

Supplementary References

1. Li, H. *et al.* The Sequence Alignment/Map format and SAMtools. *Bioinforma. Appl. NOTE* **25**, 2078–2079 (2009).

2. Link, V. *et al.* ATLAS: Analysis Tools for Low-depth and Ancient Samples. *bioRxiv* 105346 (2017). doi:10.1101/105346

3. Moreno-Mayar, J. V. *et al.* Early human dispersals within the Americas. *Science* **362**, (2018).

4. Sikora, M. *et al.* Ancient genomes show social and reproductive behavior of early Upper Paleolithic foragers. *Science* **358**, 659–662 (2017).

5.).

7. Broushaki, F. *et al.* Early Neolithic genomes from the eastern Fertile Crescent. *Science* **353**, 499–503 (2016).

8. Jun, G., Wing, M. K., Abecasis, G. R. & Kang, H. M. An efficient and scalable analysis framework for variant extraction and refinement from population scale DNA sequence data.

Genome Res. **25**, gr.176552.114 (2015).

9. Malaspinas, A. S. *et al.* bammds: a tool for assessing the ancestry of low-depth whole-genome data using multidimensional scaling (MDS). *Bioinformatics* **30**, 2962–2964 (2014).
10. Valdiosera, C. *et al.* Four millennia of Iberian biomolecular prehistory illustrate the impact of prehistoric migrations at the far end of Eurasia. *Proc. Natl. Acad. Sci. U. S. A.* **115**, 3428–3433 (2018).
11. Lazaridis, I. *et al.* Ancient human genomes suggest three ancestral populations for present-day Europeans. *Nature* **513**, 409–413 (2014).
12. Cassidy, L. M. *et al.* Neolithic and Bronze Age migration to Ireland and establishment of the insular atlantic genome. *Proc. Natl. Acad. Sci. U. S. A.* **113**, 368–373 (2016).
13. Günther, T. *et al.* Population genomics of Mesolithic Scandinavia : Investigating early postglacial migration routes and high-latitude adaptation. *PLoS Biol.* **16**, e2003703 (2018).
14. Gamba, C. *et al.* Genome flux and stasis in a five millennium transect of European prehistory. *Nat. Commun.* **5**, 1–9 (2014).
15. Ebenesersdóttir, S. S. *et al.* Ancient genomes from Iceland reveal the making of a human population. *Science* **360**, 1028–1032 (2018).
16. Margaryan, A. *et al.* Population genomics of the Viking world. *Nature* **585**, 390–396 (2020).
17. Amorim, C. E. G. *et al.* Understanding 6th-century barbarian social organization and migration through paleogenomics. *Nat. Commun.* **9**, 3547 (2018).
18. Schlebusch, C. M. *et al.* Southern African ancient genomes estimate modern human divergence to 350,000 to 260,000 years ago. *Science* **358**, 652–655 (2017).
19. Lipson, M. *et al.* Ancient West African foragers in the context of African population history. *Nature* **577**, 665–670 (2020).
20. Gallego Llorente, M. *et al.* Ancient Ethiopian genome reveals extensive Eurasian admixture throughout the African continent. *Science* **350**, 820–822 (2015).
21. Jones, E. R. *et al.* Upper Palaeolithic genomes reveal deep roots of modern Eurasians. *Nat. Commun.* **6**, 1–8 (2015).
22. de Barros Damgaard, P. *et al.* The first horse herders and the impact of early Bronze Age

steppe expansions into Asia. *Science* **360**, (2018).

23. Fu, Q. *et al.* Genome sequence of a 45,000-year-old modern human from western Siberia. *Nature* **514**, 445–449 (2014).

24. Sikora, M. *et al.* The population history of northeastern Siberia since the Pleistocene. *Nature* **570**, 182–188 (2019).

25.).
