# Supplementary material for: Imputation of ancient human genomes
Source: Nat Commun. 2023 Jun 20;14:3660. doi: 10.1038/s41467-023-39202-0 (PMC10282092; doi:10.1038/s41467-023-39202-0)
Supplement: Supplementary file 3 — Reporting Summary [file 41467_2023_39202_MOESM3_ESM.pdf]

nature portfolio

Corresponding author(s): Anna-Sapfo Malaspinas
Olivier Delaneau

Last updated by author(s): Jun 8, 2023

Reporting Summary

Nature Portfolio wishes to improve the reproducibility of the work that we publish. This form provides structure for consistency and transparency in reporting. For further information on Nature Portfolio policies, see our Editorial Policies and the Editorial Policy Checklist.

Statistics

For all statistical analyses, confirm that the following items are present in the figure legend, table legend, main text, or Methods section.

n/a	Confirmed	
☐	☒	The exact sample size (*n*) for each experimental group/condition, given as a discrete number and unit of measurement
☒	☐	A statement on whether measurements were taken from distinct samples or whether the same sample was measured repeatedly
☐	☒	The statistical test(s) used AND whether they are one- or two-sided<br>*Only common tests should be described solely by name; describe more complex techniques in the Methods section.*
☒	☐	A description of all covariates tested
☐	☒	A description of any assumptions or corrections, such as tests of normality and adjustment for multiple comparisons
☐	☒	A full description of the statistical parameters including central tendency (e.g. means) or other basic estimates (e.g. regression coefficient) AND variation (e.g. standard deviation) or associated estimates of uncertainty (e.g. confidence intervals)
☐	☒	For null hypothesis testing, the test statistic (e.g. F, t, r) with confidence intervals, effect sizes, degrees of freedom and P value noted<br>*Give P values as exact values whenever suitable.*
☒	☐	For Bayesian analysis, information on the choice of priors and Markov chain Monte Carlo settings
☒	☐	For hierarchical and complex designs, identification of the appropriate level for tests and full reporting of outcomes
☐	☒	Estimates of effect sizes (e.g. Cohen's d, Pearson's r), indicating how they were calculated

Our web collection on statistics for biologists contains articles on many of the points above.

Software and code

Policy information about availability of computer code

Data collection	No software was used for the data collection of this study.

Data analysis

Publicly available code used in this study :
SAMtools v1.10 (based on HTSlib v1.10)
BCFtools v1.10 (based on HTSlib v1.10)
ATLAS v0.9.9 (https://bitbucket.org/wegmannlab/atlas/wiki/Home)
BamUtil v1.0.14 (https://genome.sph.umich.edu/wiki/BamUtil)
GLIMPSE v1.1.1 (https://odelaneau.github.io/GLIMPSE/)
Beagle v4.1 (https://faculty.washington.edu/browning/beagle/b4_1.html)
PLINK v1.90 and PLINK v2.0
eigensoft v7.2.1 (https://github.com/DReichLab/EIG)
ADMIXTURE v1.3.0 (https://dalexander.github.io/admixture/)
Picard liftoverVCF v2.18.11 (https://gatk.broadinstitute.org/hc/en-us/articles/360037060932-LiftoverVcf-Picard-)
Picard v1.127
GATK v3.3.0
R v4.0.3
Python v3.6.12
bamdamage (part of bammds, https://github.com/sneuensc/mapache)

Downsampling, imputation and post-imputation imputation filtering scripts are deposited on github: https://github.com/bsmota/aDNA_imputation

For manuscripts utilizing custom algorithms or software that are central to the research but not yet described in published literature, software must be made available to editors and reviewers. We strongly encourage code deposition in a community repository (e.g. GitHub). See the Nature Portfolio guidelines for submitting code & software for further information.

Data

Policy information about availability of data

All manuscripts must include a data availability statement. This statement should provide the following information, where applicable:

- Accession codes, unique identifiers, or web links for publicly available datasets
- A description of any restrictions on data availability
- For clinical datasets or third party data, please ensure that the statement adheres to our policy

The Koszyce ancient trio data (RISE1159, RISE1160, RISE1168) generated in this study have been deposited in the European Nucleotide Archive (ENA) database under accession code PRJEB61632 (https://www.ebi.ac.uk/ena/browser/view/PRJEB61632).

The unfiltered imputed ancient genomes are available on Zenodo (https://doi.org/10.5281/zenodo.7993392).

The 1000 Genomes Project phase 3: 30X coverage whole genome sequencing data is available at the European Nucleotide Archive, under project PRJEB31736 and secondary study accession ERP114329 (https://www.ebi.ac.uk/ena/browser/view/PRJEB31736).

The Simons Genome Diversity Project (SGDP) bam files aligned to hg19 reference genome were downloaded from Seven Bridges Cancer Genomics Cloud.

The Allen Ancient DNA Resource (AADR) (that we refer to as the 1240K dataset) is publicly available at https://reich.hms.harvard.edu/allen-ancient-dna-resource-aadr-downloadable-genotypes-present-day-and-ancient-dna-data .

The 40 publicly available ancient human genomes in this study have origin on the following studies:
atp016: Valdiosera et al., PNAS (2018) (https://doi.org/10.1073/pnas.1717762115)
Stuttgart & Loschbour: Lazaridis et al., Nature (2014) (https://doi.org/10.1038/nature13673)
Ballynahatty & Rathlin1: Cassidy et al., PNAS (2016) (https://doi.org/10.1073/pnas.151844511)
sf12: Günther et al., PloS Biology (2018) (https://doi.org/10.1371/journal.pbio.2003703)
NE1 & BR2: Gamba et al., Nat. Com. (2014) (https://doi.org/10.1038/ncomms6257)
SIII: Sikora et al., Science (2017) (https://doi/10.1126/science.aao1807)
SSG-A-2, HSJ-A-1 & STT-A-2, Ebenesersdottir et al., Science (2018) (https://doi/10.1126/science.aar2625)
VK1: Margaryan et al., Nature (2020) (https://doi.org/10.1038/s41586-020-2688-8)
SZ15, SZ3, SZ4, SZ45, SZ43 & SZ1: Amorim et al., Nat. Com. (2018) (https://doi.org/10.1038/s41467-018-06024-4)
baa01, ela01 & new01: Schlebusch et al., Science (2017) (https://doi/10.1126/science.aao6266)
I10871: Lipson et al., Nature (2020) (https://doi.org/10.1038/s41586-020-1929-1)
Mota: Gallego Llorente et al., Science (2015) (https://doi/10.1126/science.aad2879)
KK1: Jones et al., Nat. Com., (2015) (https://doi.org/10.1038/ncomms9912)
WC1: Broushaki et al., Science (2016) (https://doi/10.1126/science.aaf7943)
BOT2016 & Yamnaya: Damgaard et al., Science (2018) (https://doi/10.1126/science.aar7711)
Andaman, AHUR_2064, Lovelock2, Lovelock3, Clovis, Sumidouro5, A460: Moreno-Mayar et al., Science (2018) (https://doi/10.1126/science.aav2621)
USR1: Moreno-Mayar et al., Nature (2018) (https://doi.org/10.1038/nature25173)
Saqqaq: Rasmussen et al., Nature (2010) (https://doi.org/10.1038/nature08835)
Ust'Ishim: Fu et al., Nature (2014) (https://doi.org/10.1038/nature13810)
Kolyma_River & Yana: Sikora et al., Nature (2019) (https://doi.org/10.1038/s41586-019-1279-z)

Human research participants

Policy information about studies involving human research participants and Sex and Gender in Research.

Reporting on sex and gender	No sex- or gender-based analyses were conducted, as we only report imputation results for the autosomes.
Population characteristics	The genetic data from all the populations in the 1000 Genomes Project 3 was used in this study.<br><br>We used a subset of 23 present-day European genomes that are part of the SGDP dataset.<br><br>We used a subset from the Allen Ancient DNA Resource (AADR) dataset that included ancient individuals labeled as Western Hunter Gatherers, Anatolian farmers and Steppe people.<br><br>The 43 ancient genomes we used in the experiments come from different continents/regions: Europe (22), Africa (5), Western Asia (4), South Asia (1), Siberia (3), America (8).
Recruitment	N/A
Ethics oversight	N/A

Note that full information on the approval of the study protocol must also be provided in the manuscript.

Field-specific reporting

Please select the one below that is the best fit for your research. If you are not sure, read the appropriate sections before making your selection.

☒ Life sciences ☐ Behavioural & social sciences ☐ Ecological, evolutionary & environmental sciences

For a reference copy of the document with all sections, see nature.com/documents/nr-reporting-summary-flat.pdf

Life sciences study design

All studies must disclose on these points even when the disclosure is negative.

Sample size	For the downsampling and imputation experiments, the sample size was 43, 42 of which had depth of coverage above 10x, which was necessary to assess imputation accuracy of low-coverage genomes, and this sample size was then defined by the availability of the data. To compare imputation accuracy of modern and present-day European genomes, we randomly selected 23 present-day European genomes in the Simons Genome Diversity Project (SGDP) to closely match the number of high-coverage ancient European genomes in our dataset (n=21). The sample size of the imputation reference panel, that is, the 1000 Genomes panel, was 2504, the total number of available non-related genomes in this dataset. For the genetic clustering analyses of ancient Europeans, we use a subset of 61 ancient genomes from the Allen Ancient DNA Resource labeled as either Western Hunter-Gatherers, or Anatolian farmers or Steppe individuals, and this sample size of 61 is limited by the data availability.
Data exclusions	Imputation was only performed at the bi-allelic SNPs for which data is available in the 1000 Genomes phase3 (high-coverage) dataset, since bi-allelic sites are the most commonly used in ancient DNA studies.<br>At the downstream analyses, in order to exclude poorly imputed variant sites, we removed rare sites, keeping minor allele sites above 1% or 5%, and removed imputed sites with genotype probability below 0.80.<br>To generate the validation dataset from the high-coverage genomes, we called genotypes and applied QC as described in Moreno-Mayar et al. (2018).
Replication	All the data and software used in this study are publicly available for the replication of the results. We deposited the code and scripts used to generate the benchmark of imputation of ancient genomes on github (https://github.com/bsmota/aDNA_imputation). We make available an ancient trio data in the European Nucleotide Archive (ENA) under accession code PRJEB61632 (https://www.ebi.ac.uk/ena/browser/view/PRJEB61632).
Randomization	No samples were split into experimental groups.
Blinding	Blinding is not relevant to this study since no group allocation occurs.

Reporting for specific materials, systems and methods

We require information from authors about some types of materials, experimental systems and methods used in many studies. Here, indicate whether each material, system or method listed is relevant to your study. If you are not sure if a list item applies to your research, read the appropriate section before selecting a response.

Materials & experimental systems

n/a	Involved in the study
☒	☐ Antibodies
☒	☐ Eukaryotic cell lines
☐	☒ Palaeontology and archaeology
☒	☐ Animals and other organisms
☒	☐ Clinical data
☒	☐ Dual use research of concern

Methods

n/a	Involved in the study
☒	☐ ChIP-seq
☒	☐ Flow cytometry
☒	☐ MRI-based neuroimaging

Palaeontology and Archaeology

Specimen provenance: All the specimens have been sampled, sequenced and published in previous studies.

Specimen deposition: N/A

Dating methods: N/A

☐ Tick this box to confirm that the raw and calibrated dates are available in the paper or in Supplementary Information.

Ethics oversight: N/A

Note that full information on the approval of the study protocol must also be provided in the manuscript.
